# Cross-Sectional Study on the Gut Microbiome of Parkinson’s Disease Patients in Central China

**DOI:** 10.3389/fmicb.2021.728479

**Published:** 2021-09-28

**Authors:** Liangwei Mao, Yu Zhang, Jing Tian, Ming Sang, Guimin Zhang, Yuling Zhou, Puqing Wang

**Affiliations:** ^1^State Key Laboratory of Biocatalysis and Enzyme Engineering, Hubei Collaborative Innovation Center for Green Transformation of Biological Resources, School of Life Sciences, Hubei University, Wuhan, China; ^2^Hubei Clinical Research Center of Parkinson’s Disease, Xiangyang No. 1 People’s Hospital, Hubei University of Medicine, Xiangyang, China

**Keywords:** Parkinson’s disease, gut-brain-axis, shotgun metagenomic sequencing, gastrointestinal dysbiosis, short-chain fatty acids

## Abstract

Gastrointestinal dysfunction plays an important role in the occurrence and development of Parkinson’s disease (PD). This study investigates the composition of the gut microbiome using shotgun metagenomic sequencing in PD patients in central China. Fecal samples from 39 PD patients (PD group) and the corresponding 39 healthy spouses of the patients (SP) were collected for shotgun metagenomics sequencing. Results showed a significantly altered microbial composition in the PD patients. *Bilophila wadsworthia* enrichment was found in the gut microbiome of PD patients, which has not been reported in previous studies. The random forest (RF) model, which identifies differences in microbiomes, reliably discriminated patients with PD from controls; the area under the receiver operating characteristic curve was 0.803. Further analysis of the microbiome and clinical symptoms showed that *Klebsiella* and *Parasutterella* were positively correlated with the duration and severity of PD, whereas hydrogen-generating *Prevotella* was negatively correlated with disease severity. The Cluster of Orthologous Groups of protein database, the KEGG Orthology database, and the carbohydrate-active enzymes of gene-category analysis showed that branched-chain amino acid–related proteins were significantly increased, and GH43 was significantly reduced in the PD group. Functional analysis of the metagenome confirmed differences in microbiome metabolism in the PD group related to short-chain fatty acid precursor metabolism.

## Introduction

Parkinson’s disease (PD) is a progressive neurodegenerative disease whose prevalence rate among central nervous system (CNS) diseases is only second to that of Alzheimer’s disease in the elderly population. The burden of PD will continue to rise with the increase in population size and aging (Castillo et al., 2019). Globally, there were 6.2 million PD patients in 2015; of these, 0.117 million patients died of PD (Mortality, 2016). Most PD cases are sporadic, and the average age at onset is 60 years (Cook Shukla et al., 1993). The pathogenesis of PD is complex, with some of the pathological features being the loss of dopaminergic neurons in the substantia nigra and intracytoplasmic inclusions (Lewy bodies) in the remaining intact nigral neurons (Braak and Braak, 2000).

Dysbiosis of the gut microbiome can affect both the enteric nervous system and the CNS. Previous studies have revealed the existence of the brain–gut–microbiome axis whose bidirectional interaction between the gut microbiome and the human nervous system can cause CNS disease (Cox and Weiner, 2018). In recent years, metagenomics studies have further revealed the correlation between PD and abnormal gut microbiome, which is also an extension of the gastrointestinal hypothesis in PD (Holmqvist et al., 2014). Gastrointestinal dysfunction, as well as changes in microbiome metabolites, can lead to inflammation, impaired epithelial barrier function, and an increase in the translocation of lipopolysaccharides and short-chain fatty acids (SCFAs), thereby causing systemic inflammatory reactions. Inflammatory cytokines pass through the blood–brain barrier to activate the microglia and astrocytes, which leads to neuroinflammation, altered cognition and behaviors, stress, and finally PD (Sampson et al., 2016; Sun and Shen, 2018). Previous studies have noted distinct alterations in the gut microbiome of PD patients, specifically SCFAs and other metabolites (Sampson et al., 2016; Unger et al., 2016; Hill-Burns et al., 2017; Sun et al., 2018).

Currently, the majority of studies use 16S rRNA amplicon sequencing technology to analyze gut microbiome diversity. However, the 16S rRNA primers used for different regions may lead to inconsistent results, because not only does the corresponding flanking conservative region have obvious binding affinity, but also the resolution of each variable region in the taxonomic group is also different (Soergel et al., 2012). Although shotgun metagenomics is expensive, it provides higher resolution and better strain identification potential, which enhances specific classification of the taxon and function (Norman et al., 2015). Only one study, conducted in Germany, has reported the use of this method in PD to date. This study produced results that could not be obtained by the use of 16S rRNA amplicon sequencing technology and revealed the differences in microbiome and microbiome metabolic pathways between L-DOPA–naive PD patients and the control group. Moreover, the study further analyzed the abundances of prophages, plasmids, and total virus (Bedarf et al., 2017). However, shotgun metagenomics has not been applied in studies of PD patients in Central China.

In China, a total of five institutions, in Beijing (Li et al., 2017c), Shanghai (Qian et al., 2018), Guangzhou (Lin et al., 2018), Changchun (Li C. et al., 2019), and Jinzhou (Li F. et al., 2019), have analyzed gut microbiome diversity using 16S rRNA amplicon sequencing technology. However, in consideration of the strong impact that geographic (Rehman et al., 2016; Kushugulova et al., 2018) and population (Deschasaux et al., 2018) factors have on the gut microbiome, shotgun metagenomics was used in this study to analyze the gut microbiome of PD populations in central China. Studies have shown that long-term habitual diets can change the structure of the gut microbiome (David et al., 2014; Krautkramer et al., 2016). Comparison of people from different families has shown that couples share a similar gut microbiome (Song et al., 2013). Therefore, in this study, PD patients’ spouses were recruited (the SP group) in order to minimize the impact of diet and living habits. Shotgun metagenomics analysis revealed significant differences in the gut microbiome composition and function between the PD patients and their spouses, further demonstrating the existence of proinflammatory dysbiosis in PD.

## Materials and Methods

### Patient Cohorts

This was a cross-sectional study, and the study subjects were recruited from the Neurology Department of Xiangyang No. 1 People’s Hospital. The study was approved by the Ethics Committee of Xiangyang No. 1 People’s Hospital. All subjects gave their consent to participate in the study in accordance with the informed consent regulations of the institution where the research was conducted. In order to reduce the potential impact of diet, daily schedule, and other related factors, all the subjects were couples in which one spouse was a PD patient (PD), whereas the other was healthy for comparison purposes (SP). PD patients were diagnosed using the PD diagnostic criteria in the movement disorder society (MDS) 2015 (Li et al., 2017a). The core standard of diagnosis was to identify if the patient had PD symptoms. The patient was considered as having PD syndromes if he/she had bradykinesia in combination with static tremor and/or muscular rigidity. Upon diagnosis with PD syndrome, further diagnosis was made based on the inclusion and exclusion criteria and warning signs to ensure that the patient was a clinical PD patient. The exclusion criteria for the experimental group based on previous literature (Keshavarzian et al., 2015) were as follows: (1) administration or infusion of antibiotics or probiotics in the recent 3 months; (2) serious disease of the gastrointestinal tract; (3) severe mental disorder; (4) too low platelet count (80 × 10^9^/L); (5) prothrombin time > 15 s; (6) History of hemorrhage in any of the visceral organs. There was no detailed dietary plan put in place for all the study subjects, and fecal samples were collected from the first defecation on the day. General demographic parameters and clinical symptoms of the study participants are shown in Table 1, while Supplementary Table 1 shows detailed patient clinical information.

**TABLE 1:** General demographic parameters and clinical manifestations.

	**PD group**	**SP group**
**Demographics**		
No. of participants	39	39
Age (years, mean ± SD)	63.95 ± 6.92	64.82 ± 6.86
Male	21	18
BMI (kg/m^2^, IQR)	23.15 (20.67–25.39)	24.2 (20.2–26.02)
**Clinical data**		
Age at onset (years, mean ± SD)	60.49 ± 6.54	–
Duration (years)		
≥3 years	21	–
>3 ≤ 5 years	11	–
>5 ≤ 10 years	6	–
>10 years	1	–
**H&Y stage**		
1	12	–
1.5	8	–
2	6	–
2.5	6	–
3	3	–
3.5	1	–
4	3	–
UPDRS-III score (mean ± SD)	34.92 ± 20.25	–

### DNA Library Construction and Sequencing Using the BGISEQ-500 Platform

DNA was extracted from the fecal sample as previously described using the MetaHIT protocol (Qin et al., 2012). Qubit (Invitrogen) was used to estimate the DNA concentration. After DNA extraction, genomic libraries were prepared following the manufacturer’s standard instructions (MGI, China). To establish a paired-end library with the insertion of 350 bp, 500 ng DNA was used, and sequencing was performed using a BGISEQ-500 sequencer through PE100 mode (Fang et al., 2018). The 1,761.8 GB original sequencing data were deposited in the Sequence Read Archive under the accession number of PRJNA588035.

### Taxonomical Analysis

All shotgun metagenomics data were handled according to the Microbiome Helper standard operating procedures (Comeau et al., 2017). FastQC tool^1^ was used to check the quality of raw reads of the metagenome. KneadData^2^ was used to trim the low-quality sequences (parameter: “SLIDINGWINDOW: 4: 20 MINLEN: 50”) and delete any unwanted human genome (HG19) reads (parameter: –very-sensitive –dovetail). The default parameters of MetaPhlAn 2.0 software (Truong et al., 2015) were used for taxonomic profiling and estimation of the reads’ abundance after processing. This software utilizes unique clade-specific marker genes to test the taxonomic clade present in the microbiome sample and estimate their relative abundance. Thus, relative abundances were multiplied by the number of sequences and rounded (Segata et al., 2011). Shannon and Chao1 indices were used to estimate α-diversity. β-Diversity was evaluated based on the Bray–Curtis dissimilarity index. The abundance of genera in all the samples was subjected to non-parametric permutational multivariate analysis of variance (PERMANOVA) to evaluate the sample cluster under various predictive factors such as disease status, gender, and age. Principal coordinate analysis (PCoA) was used to visualize the data. For PERMANOVA analysis, we used the “adonis” procedure in the vegan 2.5–4 package. Linear discriminant analysis (LDA) effect size (LEfSe) was used to identify biomarkers in the two groups. Only the taxa with *p* < 0.05 (Kruskal–Wallis test) and LDA score > 2 were considered to show statistically significant enrichment.

SeqKit software was used to convert fastq format to fasta format in order to evaluate the number of phages and plasmids (Bedarf et al., 2017). Further, the sensitive mode of Diamond software was used to map the reads to the ACLAME database (Leplae et al., 2010) after kneadData processing. Among them, the mapping reads with an *e* value < 1e-7 were considered to be valid hits. The reads’ quantity of each sample mapped to the plasmid and phage database was divided by clean reads to ensure standardization. The Mann–Whitney *U* test was used to test for the differences between groups in the R environment.

### Enterotype Analysis

An enterotype is the objective aggregation effect of gut microbiome, which is presented in the high-dimensional feature space and is another general measure of the gut microbiome (Arumugam et al., 2011; Ding and Schloss, 2014). Jensen–Shannon divergence (JSD) distances and Partitioning Around Medoids (PAM) clustering algorithm were used for cluster analysis of all the samples based on the relative abundance of genera. The Calinski–Harabasz (CH) index was used to evaluate the optimal cluster number. The χ^2^ test was used to explore whether the distribution of enterotypes was influenced by disease status.

### Gut Microbiome–Clinical Manifestation Correlation Analysis

Spearman correlation between the relative abundance of genera and clinical manifestation with more than 40% distribution among all patients was tested to evaluate the relationship between the gut microbiome and clinical manifestation. Only the genus with a clinical manifestation correlation parameter of *p* < 0.1 and ρ > 0.25 was used for visualization. This step was realized in the R environment, and the packages used included ggstatsplot (0.0.10), data.table (1.12.2), dplyr (0.8.0.1), tidyr (0.8.3), and ggplot2 (3.1.1).

### Establishment of the Disease Classification Model

The RF model was established based on the relative abundance of genera of the gut microbiome in all subjects in order to confirm the features of fecal bacteria for disease classification of the metagenome samples. Leave-one-out cross-validation was used to verify the accuracy of the model (Bedarf et al., 2017; Wang et al., 2019). The number of decision trees in the RF was set to be 5,000 (ntree = 5,000), whereas the number of preselected features at each tree node was determined as the square root of the number of features minus one, and the seed was set to be 2,019,613. The variable with the strongest classification capacity was determined based on the mean decrease accuracy (MDA), and the RF model was established. Receiver operating characteristic (ROC) curve was developed, and the areas under the curve (AUCs) of ROC were calculated to evaluate the accuracy of the new criteria for disease prediction. The work was completed in R (4.6-14, RF package).

### Gene Catalog Construction and Differential Gene Analysis

The assembly software MEGAHIT (v1.2.9) (Li et al., 2015) based on the principle of De Bruijn graphs was used to assemble the quality-filtered metagenomic sequences of each sample, and the obtained contigs were evaluated by QUAST (v5.0.2) (Gurevich et al., 2013). The prediction of protein-coding genes was performed using Prokka (v1.13) software (Seemann, 2014) with the “—metagenome –kingdom Archaea, Bacteria, Viruses” option. The open reading frame (ORF) of each sample was clustered using CD-HIT tool (v4.8.1) (Li et al., 2001) with parameters “-aS 0.9 -c 0.95 -G 0 -M 0 -T 9 -g 1” in order to obtain an initial non-redundant gene catalog (nrGC) with 95% sequence identity and 90% coverage. Unigene annotation was performed by running Diamond against the eggNOG database (Huerta-Cepas et al., 2019) and dbCAN2 database (Zhang et al., 2018). Salmon (v1.3.0) (Patro et al., 2017) was used to determine the relative abundances of genes in each sample successfully mapped to the initial nrGC. Protein abundances were quantified as counts per million, calculated by the raw valid counts (number of valid alignments) divided by the library sizes and multiplied by one million. Statistical analyses were performed using Statistical Analysis of Metagenomic Profiles (STAMP) (Parks et al., 2014).

### Pathway Analysis

Compared with taxology analysis, functional analysis aims to quantify metabolic pathways contributed by known and characterless microbiome members (Franzosa et al., 2015). HMP Unified Metabolic Analysis Network (HUMAnN2) (Franzosa et al., 2018) directly determines the gene family abundance, metabolic pathway abundance, and metabolic pathway coverage of each sample from the read after preliminary treatment. In this study, emphasis was placed on analysis in the output of metabolic pathway abundance. STAMP software was used to identify the pathways for which statistical differences existed between groups (Parks et al., 2014). Welch *t* test was used to compare cases versus controls with a Storey FDR < 0.1 as a cutoff for significance.

## Results

### Quality Metrics of Metagenomics Data

The gut microbiome of the two groups was analyzed and compared by shotgun metagenomics. After trimming and filtration using kneadData software, more than 4.05 × 10^9^ 100-bp high-quality paired-end reads were obtained, among which the total number of human reads was 4.52 × 10^7^, accounting for 1.12%. After elimination of host contamination, the average number of reads per PD patient was 5.31 × 10^7^ ± 1.58 × 10^7^, and that for the SP group was 4.95 × 10^7^ ± 2.26 × 10^7^ (Mann–Whitney *U* test, *p* = 0.24) (Supplementary Table 2). The average number of host reads in the PD patients was 6.07 × 10^5^ ± 1.01 × 10^6^, and that of the SP group was 5.53 × 10^5^ ± 1.71 × 10^6^ (Mann–Whitney *U* test, *p* = 0.03) (Supplementary Table 2). Significant increases in host reads in PD patients may reflect alterations in intestinal permeability and pathological status.

The relative abundances in the gut microbiome were measured for each sample by MetaPhlan2. The complete information for the taxonomic levels is provided in Supplementary Table 3. The majority of the reads of the tested sample of the PD group and SP group were 98.61% ± 5.45% and 99.87% ± 0.41% (Mann–Whitney *U* test, *p* = 0.67), respectively, which were all mapped to the kingdom Bacteria. The ratio corresponding to the kingdom Virus was less than that for the kingdom Bacteria: 1.36% ± 5.42% in the PD group and 0.13% ± 0.41% in the SP group (Mann–Whitney *U* test, *p* = 0.89), whereas the kingdoms Archaea and Eukaryota were almost non-existent in the samples (Supplementary Table 3).

### Differences in the Gut Microbiome

**FIGURE 1:**
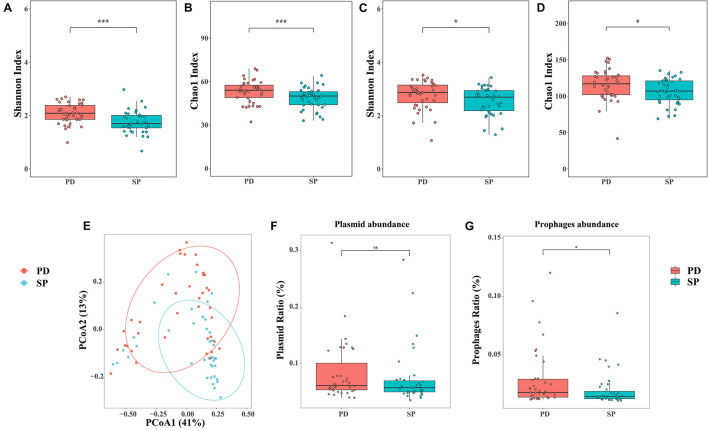
Diversity analysis and quantity of phages and plasmids. **(A,B)** Shannon and Chao1 indices of the two cohorts at the genus level. **(C,D)** Shannon and Chao1 indices of the two cohorts at the species level. **(E)** PCoA analysis of Bray–Curtis dissimilarity index between samples at the genus level. The percentage of diversity explained by each axis is indicated on the figure. **(F,G)** Relative abundance of phages and plasmids between two groups. Asterisk represents statistical significance (*p < 0.05, ***p < 0.01).

In this study, the microbiome Shannon and Chao1 indices were all analyzed at the genus and species levels, respectively. The genus Shannon (Mann–Whitney *U* test, *p* = 0.0002287) and Chao1 (Mann–Whitney *U* test, *p* = 0.007892) indices of the PD group were significantly higher than those for the SP group (Figures 1A,B). At the species level, the Shannon (Mann–Whitney *U* test, *p* = 0.02734) and Chao1 (Mann–Whitney *U* test, *p* = 0.0245) indices also showed similar trends (Figures 1C,D). The results revealed that the diversity of the gut microbiome in the PD patients was significantly higher than in the healthy group. Therefore, a higher gut microbiome Shannon and Chao1 indices may not be indicative of a healthy gut microbiome, but rather of an overgrowth of pathogenic bacteria in PD patients.

β-Diversity was analyzed at the genus level based on the disease, age, and gender status in order to estimate community diversity between the samples, using PERMANOVA. Further, the relationship between the factors and gut microbiome composition was analyzed. Between-group differences were visualized in the PCoA using the Bray–Curtis dissimilarity. The results showed that the disease status was related to the change in the between-group gut microbiome. PCoA further revealed the separation between the healthy SP group and PD group. The resolution of the top two principal coordinates was 41.40 and 13.83%, respectively (Figure 1E). However, the effects of age and gender were independent (Supplementary Table 4). This implied that the gut microbiome dysbiosis in PD patients was mainly caused by the disease itself and was not related to age and gender.

All the clean reads were mapped to the ACLAME database and standardized to estimate the known mobile elements in the metagenome. There were no significant differences in plasmid abundance between the PD and SP groups (0.083 ± 0.052 vs. 0.073 ± 0.050, Mann–Whitney *U* test, *p* = 0.8223, Figure 1F). However, phage abundance in the PD patients was higher (0.029 ± 0.025 vs. 0.020 ± 0.014, Mann–Whitney *U* test, *p* = 0.01579, Figure 1G).

### Taxonomic Changes in the Gut Microbiome

The gut microbiome was found to be mainly composed of three phyla, namely, *Bacteroidetes* (PD was 54.79% ± 16.42%, SP was 61.49% ± 12.88%, Mann–Whitney *U* test, *p* = 0.09), *Firmicutes* (PD was 28.90% ± 14.76%, SP was 30.34% ± 13.17%, Mann–Whitney *U* test, *p* = 0.47), and *Proteobacteria* (PD was 12.34% ± 17.36%, SP was 7.04% ± 6.82%, Mann–Whitney *U* test, *p* = 0.43) (Supplementary Figure 1). Two phyla that were present in small proportions, namely, *Actinobacteria* (PD was 1.54% ± 2.11%, SP was 0.56% ± 0.77%, Mann–Whitney *U* test, *p* = 0.01) and *Synergistetes* (PD was 2.52% ± 7.26%, SP was 0.33% ± 1.12%, Mann–Whitney *U* test, *p* = 0.01), exhibited significant differences, and the abundance in the PD group was significantly increased. These results showed that there were differences in the gut microbiome of the PD and SP groups at a high taxonomic level and that at a lower taxonomic level, corresponding changes could also be observed.

**FIGURE 2:**
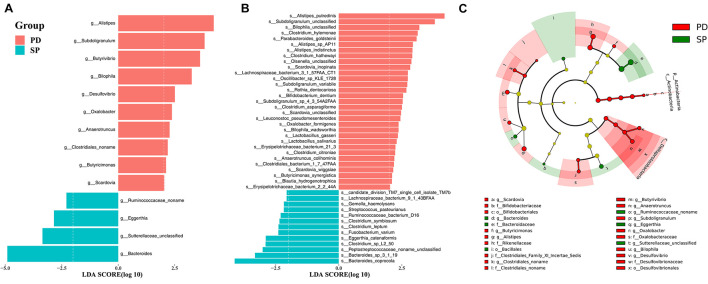
The stool microbiota profile in PD and SP groups. Differential abundance of genera **(A)** and species **(B)** between PD and SP groups identified by LEfSe. **(C)** Cladogram of the gut microbial taxa associated with PD and SP; PD-enriched taxa are in red, and SP-enriched taxa are in green.

A total of 71 taxa were identified to have notable differences between groups. The LEfSe algorithm revealed differences in 1 phylum, 2 classes, 3 orders, 7 families, 14 genera, and 44 species; details are provided in Supplementary Table 5. The enrichment at the genus and species level is shown in Figures 2A,B, respectively. In the PD group, phylum *Actinobacteria*, class *Actinobacteria*, order *Bifidobacteriales*, family *Bifidobacteriaceae*, and genus *Scardovia* were enriched at different taxonomic levels in the same clade. In addition, class *Deltaproteobacteria*, order *Desulfovibrionales*, family *Desulfovibrionaceae*, genus *Desulfovibrio*, and genus *Bilophila* also showed consistent enrichment in the PD group (Figure 2C). In the SP group, family *Bacteroidaceae* and genus *Bacteroides* shared the same clade and showed a similar trend of enrichment (Figure 2C).

### Enterotype Analysis

**FIGURE 3:**
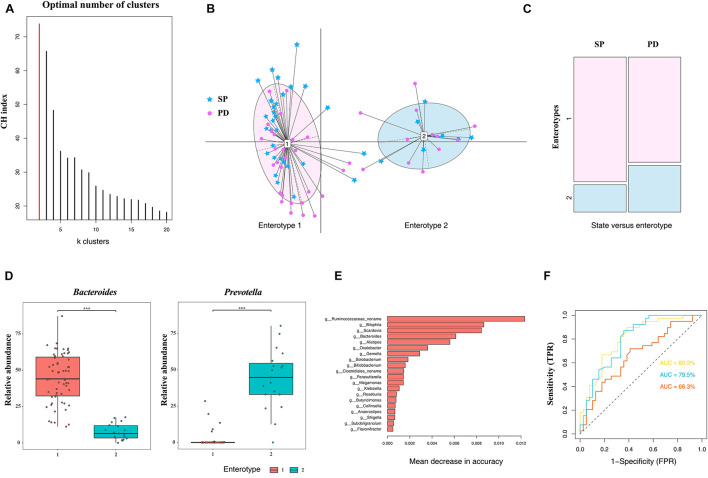
Difference in community types and taxonomic biomarkers for judging health status between PD and SP. **(A)** CH index indicates optimal classification into two community types. **(B)** Plot for PCoA of enterotypes. **(C)** Distribution of the healthy control and PD patient samples in the two enterotypes. **(D)** Relative abundances of *Bacteroides* and *Prevotella* in each enterotype. **(E)** Top 20 MDA genera were identified by applying RF classifier. **(F)** The ROC curve for predicting the occurrence of PD in 78 samples. The genus abundance profile can be used to identify microbiota samples extracted from PD patients. The dotted line represents the performance of the probabilistic model, the yellow line represents the performance of top 20 MDA variables model, the blue line represents the performance of the top 10 MDA variables model, and the orange line represents the performance of all variables. Asterisk represents statistical significance (***p < 0.01).

The classification of microbiome structures based on enterotypes has potential clinical significance. The existence of discrete enterotypes widely impacts on the study of microbiome-related human diseases. To date, studies have reported a phenotypic correlation between enterotypes (or major drive species) and human diseases (Qureshi and Mehler, 2013; Li et al., 2017b; Castano-Rodriguez et al., 2018; Cheng and Ning, 2019). Based on the microbiome, individualized diagnosis and treatment can be easily provided if patients are grouped according to enterotypes. In this study, all samples were divided into two clusters using the PAM clustering method (Figure 3A), and each cluster comprised PD patients and the control group (Figure 3B). The differences in enterotype discreteness between the patient and control were not statistically significant (Figure 3C, χ^2^ test, *p* = 0.1872). Enterotype 1 was dominated by genus *Bacteroides*, whereas enterotype 2 was dominated by the genus *Prevotella* (Figure 3D). Therefore, the enterotypes studied in PD should be further investigated in a larger patient population.

### Parkinson’s Disease Differentiation Based on the Composition of the Gut Microbiome

The RF algorithm was used to classify samples and establish a diagnostic model. One of the advantages of the RF model is that it can estimate the importance of each feature and the identification of the most important features in the classification process. Based on the measurement of the MDA, the five most important genera in the RF model were *Bilophila*, *Scardovia*, *Bacteroides*, *Alistipes*, and a novel unclassified genus of the family *Ruminococcaceae*. To improve the RF classifier results, the top 10 and 20 MDA variables were used as features to establish the model (Figure 3E). ROC curve and AUC were used to evaluate the performance of the binary classifier. We were able to distinguish the PD from the SP with AUC of 0.677 using all genera, whereas the AUC were 0.795 and 0.803 using the top 10 and 20 MDA variables, respectively, which improved diagnostic accuracy (Figure 3F). Therefore, based on these findings, differences in microbiome compositions can enable PD classification; furthermore, these can serve as biomarkers in PD diagnosis, prognosis, and therapeutic evaluation in central China.

### Correlation Between the Relative Abundance of Genera and Clinical Manifestation

**FIGURE 4:**
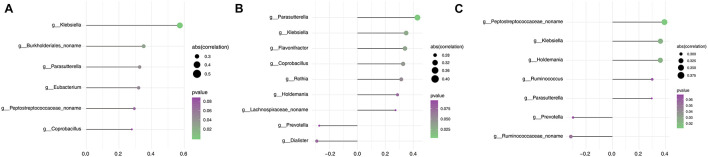
Assessment of the correlation between gut microbiota and clinical phenotype in the PD group. **(A)** Correlation analysis between PD duration and the relative abundance of genera. **(B)** Correlation analysis between H&Y stage and the relative abundance of genera. **(C)** Correlation analysis between the UPDRS III score and the relative abundance of genera. The size of node represents the degree of correlation; the gradient ramp from purple to green represents the *p* value from larger to small values.

Parkinson’s disease clinical manifestations including PD duration and disease severity (UPDRS III and H&Y stage) were quantified in correlation analysis with gut microbiome composition. Based on the Spearman correlation matrix, the correlation between clinical manifestation and relative abundance in 57 genera in which the distribution of the tested samples was greater than 40% was validated. PD duration was positively correlated to *Klebsiella* (*R*^2^ = 0.573, *p* = 0.0001); a novel unclassified genus of the family *Burkholderiales* (*R*^2^ = 0.351, *p* = 0.028), *Parasutterella* (*R*^2^ = 0.327, *p* = 0.042), and *Eubacterium* (*R*^2^ = 0.321, *p* = 0.047); and a novel unclassified genus of the family *Peptostreptococcaceae* (*R*^2^ = 0.294, *p* = 0.069) and *Coprobacillus* (*R*^2^ = 0.279, *p* = 0.086) (Figure 4A). Hoehn and Yahr (H&Y) stage showed a positive correlation with *Parasutterella* (*R*^2^ = 0.430, *p* = 0.006), *Klebsiella* (*R*^2^ = 0.349, *p* = 0.030), *Flavonifractor* (*R*^2^ = 0.341, *p* = 0.034), *Coprobacillus* (*R*^2^ = 0.327, *p* = 0.042), *Rothia* (*R*^2^ = 0.314, *p* = 0.052), and *Holdemania* (*R*^2^ = 0.287, *p* = 0.076); a novel unclassified genus of the family *Lachnospiraceae* (*R*^2^ = 0.273, *p* = 0.093); and a negative correlation with *Dialister* (*R*^2^ = -0.291, *p* = 0.073) and *Prevotella* (*R*^2^ = -0.273, *p* = 0.093) (Figure 4B). UPDRS III score showed a positive correlation with a novel unclassified genus of the family *Peptostreptococcaceae* (*R*^2^ = 0.392, *p* = 0.013), *Klebsiella* (*R*^2^ = 0.362, *p* = 0.023), *Holdemania* (*R*^2^ = 0.361, *p* = 0.024), *Ruminococcus* (*R*^2^ = 0.300, *p* = 0.064), and *Parasutterella* (*R*^2^ = 0.296, *p* = 0.067) and a negative correlation with a novel unclassified genus of the family *Ruminococcaceae* (*R*^2^ = -0.315, *p* = 0.0515) and *Prevotella* (*R*^2^ = -0.298, *p* = 0.065) (Figure 4C). Based on these findings, *Klebsiella* and *Parasutterella* showed a positive correlation with PD duration and disease severity, whereas *Prevotella* showed a negative correlation with disease severity.

### Significant Difference in Protein Abundance Between the Two Groups

**FIGURE 5:**
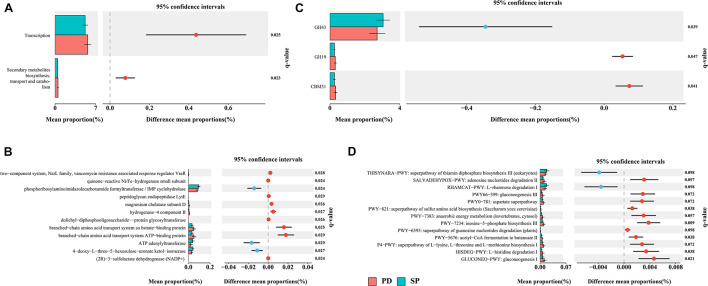
Differentially abundant proteins and functional alterations between PD and SP. **(A)** Differences in relative abundances of COG functional categories. **(B)** Differences in relative abundances of KO functional categories. Only *q* values (false discovery rate–adjusted *p* value) of <0.03 are shown. **(C)** Differences in relative abundances of CAZyme functional categories. **(D)** Metagenomic profile comparisons of differentially abundant pathways between PD and SP group samples; features with *q* < 0.1 were considered significant and were thus retained. A positive difference between proportions denotes higher abundance in PD (red), whereas a negative difference between proportions shows higher abundance in SP (blue).

This study aims to bridge the gap between previous 16S rRNA sequencing studies and functional studies by using high-resolution shotgun metagenomic sequencing to identify gut microbiome taxonomic and functional profiles. Given the high diversity between individuals, we performed *de novo* assembly on each sample independently. The data show that the total contig length is 14.24 Gb. The N50 of the PD group was 4,604 ± 1,596 bp, and the N50 of the SP group was 4,723 ± 1,782 bp (Supplementary Table 6). There was no statistical difference in the assembly results between the two groups (Mann–Whitney *U* test, *p* = 0.9761). The final non-redundant gut gene set in this study contained 3,365,331 ORFs, among which more than 100 amino acids accounted for 72.79%. All unigenes were aligned to the EggNOG Database and dbCAN2 to classify the functions of the predicted unigenes. Among them, 2,101,873(62.46%), 1,364,199(40.54%), and 301,563(8.96%) unigenes were annotated according to the Cluster of Orthologous Groups (COG) of protein database, KEGG Orthology (KO) database, and dbCAN2 database, respectively. STAMP was used to evaluate the relative enrichment of COG, KO, and carbohydrate-active enzymes (CAZymes) gene categories between the PD and control metagenomes. Enrichment analysis of COG-annotated proteins shows that transcription and secondary metabolite biosynthesis, transport, and catabolism were significantly higher in the PD patient group (Figure 5A). Based on the findings for the KO annotated proteins, 86 proteins were found to be significantly different between PD patients and controls (Figure 5B). In regard to CAZymes, the study found that GH43 was significantly reduced, whereas GH19 and CBM51 were significantly higher in PD patients (Figure 5C). The complete information is provided in Supplementary Table 7.

### Pathway Analysis

A total of 474 biologically specific pathways (Supplementary Table 7) were identified in the two sample sets using the MetaCyc database in order to determine the differences in metabolic potential of the gut microbiome between PD patients and controls. The majority of the pathways were related to bacteria, and this was similar to the taxon results in MetaPhlAn2. PERMANOVA analysis based on the Bray–Curtis dissimilarity index showed that when the health status was used as the grouping variable, the difference in abundance of metabolic pathways distinguished the PD group from the SP group (*P* = 0.063, *R*^2^ = 0.039). In STAMP, 13 metabolic pathways were identified to have significant differences between the PD and SP groups (Welch *t* test, storey FDR *q* < 0.1) (Figure 5D). The L-rhamnose degradation I (RHAMCAT-PWY) and superpathway of thiamin diphosphate biosynthesis III (THISYNARA-PWY) were found to be enriched in the gut microbiome in the SP group, whereas gluconeogenesis I (GLUCONEO-PWY); L-histidine degradation I (HISDEG-PWY); superpathway of L-lysine, L-threonine, and L-methionine biosynthesis I (P4-PWY); acetyl-CoA fermentation to butanoate II (PWY-5676); superpathway of guanosine nucleotides degradation (PWY-6595); inosine-5′-phosphate biosynthesis III (PWY-7234); anaerobic energy metabolism (PWY-7383); superpathway of sulfur amino acid biosynthesis (PWY-821); aspartate superpathway (PWY0-781); gluconeogenesis III (PWY66-399); and adenosine nucleotides degradation II (SALVADEHYPOX-PWY) were found to be enriched in the PD group. These results show that there were significant differences in the generation of precursor metabolites and energies between the two groups and also in the biological synthesis of amino acids.

## Discussion

### Main Findings

The gut microbiome is a vast and complex miniecosystem established in the human intestinal tract. It generates various metabolites that significantly affect the physiology, steady state of energy, inflammatory processes, and immunologic functions of the host, playing an important role in maintaining host health (de Clercq et al., 2016; Clavel et al., 2017; Espinoza and Minami, 2018). The advent of high-throughput sequencing technology has completely changed the understanding of the relationship between the gut microbiome and human health. In this study, the shotgun metagenomic sequencing method is used for the first time to analyze the gut microbiome in PD patients and their healthy spouses in central China. The findings reveal significant differences in the composition and function of the gut microbiome between the PD patients and their spouses. β-Diversity analysis identifies significant differences between the PD and SP groups, revealing that the differences in the gut microbiome are caused by disease status. Moreover, the bacteriophage abundance in the PD group is significantly higher than in the SP group. Therefore, the gut microbiome compositions in the PD and SP groups show significant differences at all taxonomic levels except at the kingdom level. Enterotype analysis, as one of the methods for the classification of microbial community structure, is likely to be used as an index for the evaluation of health status in the future (Hildebrand et al., 2013; Costea et al., 2018). However, no significant dispersion trend was observed between the two groups. At the genus level, the RF model, after selection of features, distinguished between the PD patients and the SP group with very high accuracy (AUC = 0.803). Correlation analysis of clinical manifestation and microbiome composition has significant value in the study of disease progression. In addition, our findings reveal differences in gene categories and microbiome metabolism, for example, branched-chain amino acid (BCAA) transport system–related proteins, GH43, and the acetyl-CoA fermentation to butanoate II pathway, which are related to the generation of precursor metabolites of SCFAs.

### Analysis of the Microbiome

Previous studies comparing the gut microbiome between PD patients and the SP group have been mainly carried out at the level of genus and above because of limitations in research methods (Li et al., 2017c; Qian et al., 2018). In this study, significant differences were identified between the composition of the gut microbiome of the PD and the SP groups at all taxonomic levels except the kingdom level. Thus, our results further strengthen the “gastrointestinal hypothesis” of PD. In the following section, we focus on the differences between the two groups at family, genus, and species levels. At the family level, the PD group showed *Bifidobacteriaceae* enrichment. This is consistent with recent studies (Scheperjans et al., 2015; Hill-Burns et al., 2017; Hopfner et al., 2017; Lin et al., 2018). No study has reported a high abundance of *Oxalobacteraceae*, *Desulfovibrionaceae*, *Rikenellaceae*, and *Clostridiales_Family_XI_Incertae_Sedis* and a novel unclassified family of the order *Clostridiales* in PD. The genera enriched in PD showed evolutionary relationships with family levels to some extent; these genera were *Desulfovibrio*, *Alistipes*, *Oxalobacter*, *Bilophila*, and *Scardovia*. In a recent study, fecal analysis of regressive infantile autism patients by pyrosequencing technology showed that *Desulfovibrio* was more common among infantile autism patients relative to the control group (Finegold, 2011). Such bacteria can generate important virulence factors that can account for several pathologic features of infantile autism. Interestingly, PD patients also suffer from depression, cognitive disorders, and other non-motor symptoms (Chen et al., 2013). The genera enriched in PD that showed no evolutionary relationship included *Butyricimonas*, *Butyrivibrio*, *Subdoligranulum*, and *Anaerotruncus*. A previous study has shown that PD patients exhibit *Ruminococcaceae* family enrichment (Li F. et al., 2019); however, this was not the case in this study. Our results show that a novel unclassified genus of the family *Ruminococcaceae* was enriched in the SP group. In addition, we found that *Bacteroides* were enriched in the control group. Study has shown that *Bacteroides* can actively improve the intestinal environment, for example, by reducing intracellular oxygen levels, thereby allowing the growth of strict anaerobes (Wexler and Goodman, 2017). Of interest is that inflammation is a central component of PD pathology. Recently, there has emerged a clear understanding that several species of *Bacteroides* express an integrase that can rapidly recruit white blood cells to kill the immune cells that cause inflammatory bowel disease and prevent the occurrence of IBD (Hebbandi Nanjundappa et al., 2017). This study further identified PD microbial markers and established a diagnostic model from the 20 MDA biomarkers with high diagnostic accuracy. Therefore, gut microbiome analysis represents a tool for the development of targeted non-invasive biomarkers for PD diagnosis.

### Key Species

It is still necessary to identify the key species in the gut microbiome that correlate with specific metabolites or disease phenotypes in order to understand the ecological interactions among them and between them and their hosts (Zhang and Zhao, 2016). We further identified enrichment of a species that has never been mentioned previously in the intestinal microbiota of PD patients, that is, *Bilophila wadsworthia*. This bacterium erodes the mucus layer of the colon, allowing bacterial entry into the lining cells (Ribaldone et al., 2018). A previous study has shown that the glycyl radical enzyme enhances the production of H_2_S by *B. wadsworthia* (Peck et al., 2019). H_2_S is highly toxicogenomic and causes ulcerative colitis and colorectal cancer (Attene-Ramos et al., 2010). This metabolite can also reduce the disulfide bond in the mucous layer of the enteric epithelium, thereby damaging the intestinal barrier (Ijssennagger et al., 2016). During aging, the increase in the permeability of the intestinal epithelium may aggravate damage to the integrity of the intestinal barrier (Camilleri et al., 2012), which could lead to PD under the combined action of several factors. Studies show that hydrogen may provide energy required for the growth of *B. wadsworthia* (da Silva et al., 2008). Thus, overgrowth of such bacteria may accelerate consumption of hydrogen in the intestinal tract. Notably, hydrogen gas selectively neutralizes toxic hydroxyl radicals, downregulates the expression of proinflammatory factors, and maintains cerebrovascular reactivity (Ostojic, 2018). Therefore, the changes in PD microbiome composition impact antioxidant processes in the gut.

### Phage Analysis

Our study shows that phages were significantly enriched in the PD group. Currently, it is not known how phages affect the structure and function of the gut microbiome in the healthy human (Manrique et al., 2016). However, some evidence indicates that phages may reflect the health status of an individual (De Sordi et al., 2019; Hsu et al., 2019). A study found that the number of phages in the intestinal tract was significantly increased in type 2 diabetes patients, and many of these phages were of an unknown type (Ma et al., 2018). Intestinal-tract phages may have various possible roles in the pathogenic mechanism underlying PD, which provides potential novel ways to elucidate the mechanisms of the gut microbiome in PD patients.

### Correlation of the Intestinal Microbiota With Clinical Manifestations of Parkinson’s Disease

In this study, some genera were related to clinical manifestations of PD, including disease duration and severity. Notably, *Klebsiella* and *Parasutterella* were positively correlated with clinical manifestations in PD, and *Prevotella* was negatively correlated with disease severity. Currently, there is no evidence supporting the relationship between hydrogen produced in the intestinal tract and PD. Some studies indicate that changes in the hydrogen-generating microbiome in PD may cause serious damage, compromising motor functions (Keshavarzian et al., 2015; Scheperjans et al., 2015). Our results also suggest that the abundance of hydrogen-generating *Prevotella* is negatively correlated with two clinical manifestations that reflect disease severity, namely, UPDRS III and H&Y stage. H_2_ is produced by intestinal microorganisms that may play a role in the pathogenesis of PD as a mediator of the brain–gut–microbiome axis (Ostojic, 2018). Although the data show that the correlation is not particularly strong, we believe that a trend exists. The above results may be due to the small number of participants, resulting in a certain degree of randomness. Larger population data are therefore needed for further research. In addition, this is a cross-sectional study, and a time-series study is required for further verification of the findings.

### Gene Enrichment Analysis

The human body carries more than 10 times the number of microbes than human cells and 100 times more microbial genes than its own (Zhu et al., 2010; Thursby and Juge, 2017). It is the gene products of these microbiota that interact with the intestinal microecosystem, and their potential functions can provide some insights into the occurrence and development of diseases. From the results of the study, it can be observed that the function class “secondary metabolites biosynthesis, transport, and catabolism” was found to be slightly enriched in PD. It is worth noting that in KO-based annotations, BCAA transport system–related proteins increased significantly in the PD group, namely, BCAA transport system ATP-binding protein, BCAA transport system permease protein, and BCAA transport system substrate-binding protein. Valine, leucine, and isoleucine are considered essential amino acids because they cannot be synthesized *de novo* and must be obtained from the diet. They participate indirectly and directly in a variety of biochemical functions in the peripheral nervous system and CNS (Fernstrom, 2005; Brosnan and Brosnan, 2006; Sperringer et al., 2017). In addition, BCAAs are considered key nitrogen donors involved in interorgan and intracellular nitrogen shuttling. Although vital for normal physiological function, excessive amounts of BCAAs are considered toxic and can cause severe tissue damage, especially to the CNS, as evidenced from the neuropathology associated with maple syrup urine disease, an autosomal recessive metabolic disorder that is caused by excessive BCAA levels (Menkes et al., 1954). Studies have shown that amino acids can be used by intestinal bacteria for the production of SCFAs and BCAAs (Elsden and Hilton, 1978). Pathway analysis results showed that the amino acid synthesis pathway was enriched in the PD group. A limitation of our study is that it is observational, limiting our ability to establish a causal relationship between BCAAs and PD. If a causal relationship between BCAAs and PD can be found, PD can be prevented in the future by modulating the dietary intake and metabolism of these amino acids.

In this study, we also found that GH43 was significantly reduced in PD patients. Studies of the human gut microbiome have identified GH43 enzymes to be among the most abundant CAZymes present (El Kaoutari et al., 2013; Wu et al., 2015). It is well known that diet is a key determinant of the structure and function of intestinal communities. Phytochemicals that enter the circulatory system may be beneficial to health through the induction of stress resistance mechanisms (autophagy, DNA repair, mitochondrial biogenesis, and expression of detoxification and antioxidant enzymes) (Martel et al., 2020). The GH43 family has emerged as important in biomass deconstruction efforts, because studies have found this family in a number of plant cell wall–degrading microorganisms (Kohler et al., 2015). Generally, the carbohydrate composition in the intestine has a profound impact on and may be one of the main driving forces shaping the composition of the intestinal microbiota. The CAZyme profile reflects the adaptability of the gut microbial communities. Therefore, we evaluated the differences in the CAZyme profiles between the two groups to further clarify the possible effects of differences in protein function on physiological functions.

### Pathways in Parkinson’s Disease

To explore dysbiosis of the microbiome according to taxonomic composition, we further analyzed metabolic pathways. Although 13 pathways differed between the two groups, this study focused more on SCFA production–related pathways. SCFAs, such as acetate, propionate, and butyrate, are products of dietary fiber fermentation by the gut microbiome and are thought to mediate microbiota–gut–brain communication (Dalile et al., 2019). They promote health by increasing the integrity of the enteric epithelium, specific antibody reactions, and the number of regulatory T cells in the colon (Arpaia et al., 2013; Smith et al., 2013). Of note is that a previous study found SCFAs to be sufficient for inducing α-syn pathology and microglial activation in α-syn–overexpressing mice (Sampson et al., 2016). Consistent with this, this study shows, for the first time, that the pathway by which acetyl coenzyme A is fermented to butyric acid II was enriched in PD patients. These results imply that at physiological concentrations in the intestinal tract, SCFAs may suppress inflammatory reactions.

### Integration of Research in China’s Mainland

Integrating research data on PD and the microbiome in five cities of China, namely, Beijing (Li et al., 2017c), Shanghai (Qian et al., 2018), Guangzhou (Lin et al., 2018), Changchun (Li C. et al., 2019), and Jinzhou (Li F. et al., 2019) (Supplementary Table 8), revealed low microbiome overlap among regions and even contradictory results. Geographical factors exert a strong effect on the human intestinal microbiota, and this partly explains the inconsistent dysbiosis patterns reported in small-scale studies in some Chinese mainland cities. However, it is important to identify microbiota associated with cross-regional consensus risk, because consistent signals can be valuable for future research in large populations. The results of the study were consistent with those of previous studies to a certain extent. This study and the Guangzhou study (Lin et al., 2018) found that, at the family level, *Desulfovibrionaceae* and *Bifidobacteriaceae* are elevated in PD patients; at the genus level, findings for *Alistipes* in the Changchun study (Li C. et al., 2019) and *Anaerotruncus* in the Shanghai study (Qian et al., 2018) are consistent with this study and have been confirmed to be elevated in PD patients. *Bacteroides* has been shown to be decreased in PD patients in both the Jinzhou study (Li F. et al., 2019) and this study. Except for the impact of geographic location, this result is not surprising, given that the human microbiome is highly heterogeneous at the genomic level and varies among individuals. Several other factors may account for the different conclusions, such as the physiological state of host, selection of study subjects, sample type, experimental method, and bioinformatics analysis. In addition, none of the studies provide information on antibiotic use in early life or previous *Clostridium difficile* infections, which are known to have profound and long-lasting effects on gut microbiota.

## Conclusion and Future Perspectives

In summary, in this study, we performed shotgun metagenomics analysis of the gut microbiome of PD patients in central China. To our knowledge, this is the first analysis of the PD gut microbiome in central China at the level of lower taxa. This study explains the changes in microbial composition, gene categories, and metabolic pathways based on analysis of fecal samples from PD patients. The principal objective of this study was to determine whether there is evidence for proinflammatory dysbiosis in PD. Further research is necessary to develop effective preventive and therapeutic strategies for PD based on microbiome manipulation.

## Data Availability Statement

The datasets presented in this study can be found in online repositories. The names of the repository/repositories and accession number(s) can be found below: https://www.ncbi.nlm.nih.gov/, PRJNA588035.

## Ethics Statement

The studies involving human participants were reviewed and approved by Ethics Committee of Xiangyang No. 1 People’s Hospital. The patients/participants provided their written informed consent to participate in this study.

## Author Contributions

LM participated in the study design, designed statistical tests to test hypothesis, interpreted bioinformatics data, and drafted the manuscript. YZ participated in the study design and analyzed and interpreted clinical data. JT and MS participated in patients’ recruitment and analyzed and interpreted clinical data. GZ participated in data analysis and drafted the manuscript. YZ participated in the study design, interpreted bioinformatics data, drafted the manuscript, and instructed and supervised this study. PW participated in the study design and patients’ recruitment, analyzed and interpreted clinical data, and instructed and supervised this study. All authors critically reviewed the article and approved the final version.

## Conflict of Interest

The authors declare that the research was conducted in the absence of any commercial or financial relationships that could be construed as a potential conflict of interest.

## Publisher’s Note

All claims expressed in this article are solely those of the authors and do not necessarily represent those of their affiliated organizations, or those of the publisher, the editors and the reviewers. Any product that may be evaluated in this article, or claim that may be made by its manufacturer, is not guaranteed or endorsed by the publisher.

## Footnotes

1
https://www.bioinformatics.babraham.ac.uk/projects/fastqc/

2
https://huttenhower.sph.harvard.edu/kneaddata

## Supplementary Material

Supplementary Figure 1 Distribution of phyla in all samples.

Supplementary Table 1 Clinical information for all PD samples.

Supplementary Table 2 Sequencing data generated for each sample.

Supplementary Table 3 Relative abundance of all taxa for each sample.

Supplementary Table 4 PERMANOVA of the influence of a single factor on the gut microbiome.

Supplementary Table 5 Details of LEfSe results between the two groups at different taxonomic levels; the LDA scores (log10) > 2 and *p* < 0.05 are listed.

Supplementary Table 6 The results of *de novo* sequence assembly of each sample.

Supplementary Table 7 Differentially abundant proteins and functional alterations between groups.

Supplementary Table 8 Altered gut microbiota composition in patients with PD observed across different studies in Chinese mainland.

## Abbreviations

AUC: area under the curve
BCAA: branched-chain amino acid
BMI: body mass index
CAZymes: carbohydrate-active enzymes
CH: Calinski–Harabasz
CNS: central nervous system
COG: Cluster of Orthologous Groups
FDR: false discovery rate
HUMAnN2: HMP unified metabolic analysis network 2
IBD: inflammatory bowel disease
IBS: irritable bowel syndrome
IBS-C: irritable bowel syndrome with constipation predominance
IBS-D: irritable bowel syndrome with diarrhea predominance
IQR: interquartile range
JSD: Jensen–Shannon divergence
KO: KEGG Orthology
LEfSe: linear discriminant analysis effect size
LOOCV: leave one out cross validation
LPS: lipopolysaccharides
MetaPhlAn2: metagenomic phylogenetic analysis v2.0
MDA: decreases in accuracy
PAM: partitioning around medoids
PCoA: principal coordinate analysis
PD: Parkinson’s disease
PERMANOVA: permutational multivariate analysis of variance
RF: random forest
ROC: receiver operating characteristic
SCFAs: short-chain fatty acids
SD: standard deviation
STAMP: statistical analysis of metagenomic profiles.

## References

[B1] Arpaia N. Campbell C. Fan X. Dikiy S. van der Veeken J. deRoos P. (2013). Metabolites produced by commensal bacteria promote peripheral regulatory T-cell generation. *Nature* 504 451–455. 10.1038/nature12726 24226773 PMC3869884

[B2] Arumugam M. Raes J. Pelletier E. Le Paslier D. Yamada T. Mende D. R. (2011). Enterotypes of the human gut microbiome. *Nature* 473 174–180. 10.1038/nature09944 21508958 PMC3728647

[B3] Attene-Ramos M. S. Nava G. M. Muellner M. G. Wagner E. D. Plewa M. J. Gaskins H. R. (2010). DNA damage and toxicogenomic analyses of hydrogen sulfide in human intestinal epithelial FHs 74 Int cells. *Environ. Mol. Mutagen* 51 304–314. 10.1002/em.20546 20120018

[B4] Bedarf J. R. Hildebrand F. Coelho L. P. Sunagawa S. Bahram M. Goeser F. (2017). Functional implications of microbial and viral gut metagenome changes in early stage L-DOPA-naive Parkinson’s disease patients. *Genome Med.* 9:39. 10.1186/s13073-017-0451-z 28449715 PMC5408370

[B5] Braak H. Braak E. (2000). Pathoanatomy of Parkinson’s disease. *J. Neurol.* 247(Suppl. 2), II3–II10. 10.1007/PL00007758 10991663

[B6] Brosnan J. T. Brosnan M. E. (2006). Branched-chain amino acids: enzyme and substrate regulation. *J. Nutr.* 136(Suppl. 1), 207S–211S. 10.1093/jn/136.1.207S 16365084

[B7] Camilleri M. Madsen K. Spiller R. Greenwood-Van Meerveld B. Verne G. N. (2012). Intestinal barrier function in health and gastrointestinal disease. *Neurogastroenterol. Motil.* 24 503–512. 10.1111/j.1365-2982.2012.01921.x 22583600 PMC5595063

[B8] Castano-Rodriguez N. Underwood A. P. Merif J. Riordan S. M. Rawlinson W. D. Mitchell H. M. (2018). Gut microbiome analysis identifies potential etiological factors in acute gastroenteritis. *Infect. Immun.* 86:e00060-18. 10.1128/IAI.00060-18 29685983 PMC6013661

[B9] Castillo X. Castro-Obregon S. Gutierrez-Becker B. Gutierrez-Ospina G. Karalis N. Khalil A. A. (2019). Re-thinking the etiological framework of neurodegeneration. *Front. Neurosci.* 13:728. 10.3389/fnins.2019.00728 31396030 PMC6667555

[B10] Chen H. Burton E. A. Ross G. W. Huang X. Savica R. Abbott R. D. (2013). Research on the premotor symptoms of Parkinson’s disease: clinical and etiological implications. *Environ. Health Perspect.* 121 1245–1252. 10.1289/ehp.1306967 23933572 PMC3855519

[B11] Cheng M. Ning K. (2019). Stereotypes about enterotype: the old and new ideas. *Genomics Proteomics Bioinformatics* 17 4–12. 10.1016/j.gpb.2018.02.004 31026581 PMC6521238

[B12] Clavel T. Gomes-Neto J. C. Lagkouvardos I. Ramer-Tait A. E. (2017). Deciphering interactions between the gut microbiota and the immune system via microbial cultivation and minimal microbiomes. *Immunol. Rev.* 279 8–22. 10.1111/imr.12578 28856739 PMC5657458

[B13] Comeau A. M. Douglas G. M. Langille M. G. (2017). Microbiome helper: a custom and streamlined workflow for microbiome research. *mSystems* 2:e00127-16. 10.1128/mSystems.00127-16 28066818 PMC5209531

[B14] Cook Shukla L. Schulze J. Farlow J. Pankratz N. D. Wojcieszek J. Foroud T. (1993). *Parkinson Disease Overview*, eds Adam M. P. Ardinger H. H. Pagon R. A. Wallace S. E. Bean L. J. H. Stephens K. (Seattle, Was: GeneReviews).

[B15] Costea P. I. Hildebrand F. Arumugam M. Backhed F. Blaser M. J. Bushman F. D. (2018). Publisher correction: enterotypes in the landscape of gut microbial community composition. *Nat. Microbiol.* 3:388. 10.1038/s41564-018-0114-x 29440750

[B16] Cox L. M. Weiner H. L. (2018). Microbiota signaling pathways that influence neurologic disease. *Neurotherapeutics* 15 135–145. 10.1007/s13311-017-0598-8 29340928 PMC5794708

[B17] da Silva S. M. Venceslau S. S. Fernandes C. L. Valente F. M. Pereira I. A. (2008). Hydrogen as an energy source for the human pathogen Bilophila wadsworthia. *Antonie Van Leeuwenhoek* 93 381–390. 10.1007/s10482-007-9215-x 18066702

[B18] Dalile B. Van Oudenhove L. Vervliet B. Verbeke K. (2019). The role of short-chain fatty acids in microbiota-gut-brain communication. *Nat. Rev. Gastroenterol. Hepatol.* 16 461–478. 10.1038/s41575-019-0157-3 31123355

[B19] David L. A. Maurice C. F. Carmody R. N. Gootenberg D. B. Button J. E. Wolfe B. E. (2014). Diet rapidly and reproducibly alters the human gut microbiome. *Nature* 505 559–563. 10.1038/nature12820 24336217 PMC3957428

[B20] de Clercq N. C. Groen A. K. Romijn J. A. Nieuwdorp M. (2016). Gut microbiota in obesity and undernutrition. *Adv. Nutr.* 7 1080–1089. 10.3945/an.116.012914 28140325 PMC5105041

[B21] De Sordi L. Lourenco M. Debarbieux L. (2019). The battle within: interactions of bacteriophages and bacteria in the gastrointestinal tract. *Cell Host Microbe* 25 210–218. 10.1016/j.chom.2019.01.018 30763535

[B22] Deschasaux M. Bouter K. E. Prodan A. Levin E. Groen A. K. Herrema H. (2018). Depicting the composition of gut microbiota in a population with varied ethnic origins but shared geography. *Nat. Med.* 24 1526–1531. 10.1038/s41591-018-0160-1 30150717

[B23] Ding T. Schloss P. D. (2014). Dynamics and associations of microbial community types across the human body. *Nature* 509 357–360. 10.1038/nature13178 24739969 PMC4139711

[B24] El Kaoutari A. Armougom F. Gordon J. I. Raoult D. Henrissat B. (2013). The abundance and variety of carbohydrate-active enzymes in the human gut microbiota. *Nat. Rev. Microbiol.* 11 497–504. 10.1038/nrmicro3050 23748339

[B25] Elsden S. R. Hilton M. G. (1978). Volatile acid production from threonine, valine, leucine and isoleucine by clostridia. *Arch. Microbiol.* 117 165–172. 10.1007/BF00402304 678022

[B26] Espinoza J. L. Minami M. (2018). Sensing bacterial-induced DNA damaging effects via natural killer group 2 member D immune receptor: from Dysbiosis to autoimmunity and carcinogenesis. *Front. Immunol.* 9:52. 10.3389/fimmu.2018.00052 29422899 PMC5788971

[B27] Fang C. Zhong H. Lin Y. Chen B. Han M. Ren H. (2018). Assessment of the cPAS-based BGISEQ-500 platform for metagenomic sequencing. *Gigascience* 7 1–8. 10.1093/gigascience/gix133 29293960 PMC5848809

[B28] Fernstrom J. D. (2005). Branched-chain amino acids and brain function. *J. Nutr.* 135(Suppl. 6), 1539S–1546S. 10.1093/jn/135.6.1539S 15930466

[B29] Finegold S. M. (2011). Desulfovibrio species are potentially important in regressive autism. *Med. Hypotheses* 77 270–274. 10.1016/j.mehy.2011.04.032 21592674

[B30] Franzosa E. A. Hsu T. Sirota-Madi A. Shafquat A. Abu-Ali G. Morgan X. C. (2015). Sequencing and beyond: integrating molecular ‘omics’ for microbial community profiling. *Nat. Rev. Microbiol.* 13 360–372. 10.1038/nrmicro3451 25915636 PMC4800835

[B31] Franzosa E. A. McIver L. J. Rahnavard G. Thompson L. R. Schirmer M. Weingart G. (2018). Species-level functional profiling of metagenomes and metatranscriptomes. *Nat. Methods* 15 962–968. 10.1038/s41592-018-0176-y 30377376 PMC6235447

[B32] Gurevich A. Saveliev V. Vyahhi N. Tesler G. (2013). QUAST: quality assessment tool for genome assemblies. *Bioinformatics* 29 1072–1075. 10.1093/bioinformatics/btt086 PMC3624806 23422339

[B33] Hebbandi Nanjundappa R. Ronchi F. Wang J. Clemente-Casares X. Yamanouchi J. Sokke Umeshappa C. (2017). A gut microbial mimic that hijacks diabetogenic autoreactivity to suppress colitis. *Cell* 171 655–667.e17. 10.1016/j.cell.2017.09.022 29053971

[B34] Hildebrand F. Nguyen T. L. Brinkman B. Yunta R. G. Cauwe B. Vandenabeele P. (2013). Inflammation-associated enterotypes, host genotype, cage and inter-individual effects drive gut microbiota variation in common laboratory mice. *Genome Biol.* 14:R4. 10.1186/gb-2013-14-1-r4 23347395 PMC4053703

[B35] Hill-Burns E. M. Debelius J. W. Morton J. T. Wissemann W. T. Lewis M. R. Wallen Z. D. (2017). Parkinson’s disease and Parkinson’s disease medications have distinct signatures of the gut microbiome. *Mov. Disord.* 32 739–749. 10.1002/mds.26942 28195358 PMC5469442

[B36] Holmqvist S. Chutna O. Bousset L. Aldrin-Kirk P. Li W. Bjorklund T. (2014). Direct evidence of Parkinson pathology spread from the gastrointestinal tract to the brain in rats. *Acta Neuropathol.* 128 805–820. 10.1007/s00401-014-1343-6 25296989

[B37] Hopfner F. Kunstner A. Muller S. H. Kunzel S. Zeuner K. E. Margraf N. G. (2017). Gut microbiota in Parkinson disease in a northern German cohort. *Brain Res.* 1667 41–45. 10.1016/j.brainres.2017.04.019 28506555

[B38] Hsu B. B. Gibson T. E. Yeliseyev V. Liu Q. Lyon L. Bry L. (2019). Dynamic modulation of the gut microbiota and metabolome by bacteriophages in a mouse model. *Cell Host Microbe* 25 803–814.e5. 10.1016/j.chom.2019.05.001 31175044 PMC6579560

[B39] Huerta-Cepas J. Szklarczyk D. Heller D. Hernandez-Plaza A. Forslund S. K. Cook H. (2019). eggNOG 5.0: a hierarchical, functionally and phylogenetically annotated orthology resource based on 5090 organisms and 2502 viruses. *Nucleic Acids Res.* 47 D309–D314. 10.1093/nar/gky1085 30418610 PMC6324079

[B40] Ijssennagger N. van der Meer R. van Mil S. W. C. (2016). Sulfide as a mucus barrier-breaker in inflammatory bowel disease? *Trends Mol. Med.* 22 190–199. 10.1016/j.molmed.2016.01.002 26852376

[B41] Keshavarzian A. Green S. J. Engen P. A. Voigt R. M. Naqib A. Forsyth C. B. (2015). Colonic bacterial composition in Parkinson’s disease. *Mov. Disord.* 30 1351–1360. 10.1002/mds.26307 26179554

[B42] Kohler A. Kuo A. Nagy L. G. Morin E. Barry K. W. Buscot F. (2015). Convergent losses of decay mechanisms and rapid turnover of symbiosis genes in mycorrhizal mutualists. *Nat. Genet.* 47 410–415. 10.1038/ng.3223 25706625

[B43] Krautkramer K. A. Kreznar J. H. Romano K. A. Vivas E. I. Barrett-Wilt G. A. Rabaglia M. E. (2016). Diet-microbiota interactions mediate global epigenetic programming in multiple host tissues. *Mol. Cell* 64 982–992. 10.1016/j.molcel.2016.10.025 27889451 PMC5227652

[B44] Kushugulova A. Forslund S. K. Costea P. I. Kozhakhmetov S. Khassenbekova Z. Urazova M. (2018). Metagenomic analysis of gut microbial communities from a Central Asian population. *BMJ Open* 8:e021682. 10.1136/bmjopen-2018-021682 30056386 PMC6067398

[B45] Leplae R. Lima-Mendez G. Toussaint A. (2010). ACLAME: a classification of mobile genetic elements, update 2010. *Nucleic Acids Res.* 38 D57–D61. 10.1093/nar/gkp938 19933762 PMC2808911

[B46] Li C. Cui L. Yang Y. Miao J. Zhao X. Zhang J. (2019). Gut microbiota differs between Parkinson’s disease patients and healthy controls in Northeast China. *Front. Mol. Neurosci.* 12:171. 10.3389/fnmol.2019.00171 31354427 PMC6637281

[B47] Li D. Liu C. M. Luo R. Sadakane K. Lam T. W. (2015). MEGAHIT: an ultra-fast single-node solution for large and complex metagenomics assembly via succinct de Bruijn graph. *Bioinformatics* 31 1674–1676. 10.1093/bioinformatics/btv033 25609793

[B48] Li F. Wang P. Chen Z. Sui X. Xie X. Zhang J. (2019). Alteration of the fecal microbiota in North-Eastern Han Chinese population with sporadic Parkinson’s disease. *Neurosci. Lett.* 707:134297. 10.1016/j.neulet.2019.134297 31200089

[B49] Li W. Wu X. Hu X. Wang T. Liang S. Duan Y. (2017c). Structural changes of gut microbiota in Parkinson’s disease and its correlation with clinical features. *Sci. China Life Sci.* 60 1223–1233. 10.1007/s11427-016-9001-4 28536926

[B50] Li J. Jin M. Wang L. Qin B. Wang K. (2017a). MDS clinical diagnostic criteria for Parkinson’s disease in China. *J. Neurol.* 264 476–481. 10.1007/s00415-016-8370-2 28025665

[B51] Li J. Zhao F. Wang Y. Chen J. Tao J. Tian G. (2017b). Gut microbiota dysbiosis contributes to the development of hypertension. *Microbiome* 5:14. 10.1186/s40168-016-0222-x 28143587 PMC5286796

[B52] Li W. Jaroszewski L. Godzik A. (2001). Clustering of highly homologous sequences to reduce the size of large protein databases. *Bioinformatics* 17 282–283. 10.1093/bioinformatics/17.3.282 11294794

[B53] Lin A. Zheng W. He Y. Tang W. Wei X. He R. (2018). Gut microbiota in patients with Parkinson’s disease in southern China. *Parkinsonism Relat. Disord.* 53 82–88. 10.1016/j.parkreldis.2018.05.007 29776865

[B54] Ma Y. You X. Mai G. Tokuyasu T. Liu C. (2018). A human gut phage catalog correlates the gut phageome with type 2 diabetes. *Microbiome* 6:24. 10.1186/s40168-018-0410-y 29391057 PMC5796561

[B55] Manrique P. Bolduc B. Walk S. T. van der Oost J. de Vos W. M. Young M. J. (2016). Healthy human gut phageome. *Proc. Natl. Acad. Sci. U.S.A.* 113 10400–10405. 10.1073/pnas.1601060113 27573828 PMC5027468

[B56] Martel J. Ojcius D. M. Ko Y. F. Young J. D. (2020). Phytochemicals as prebiotics and biological stress inducers. *Trends Biochem. Sci.* 45 462–471. 10.1016/j.tibs.2020.02.008 32413323

[B57] Menkes J. H. Hurst P. L. Craig J. M. (1954). A new syndrome: progressive familial infantile cerebral dysfunction associated with an unusual urinary substance. *Pediatrics* 14 462–467. 13214961

[B58] Mortality G. B. D. (2016). Causes of death C. Global, regional, and national life expectancy, all-cause mortality, and cause-specific mortality for 249 causes of death, 1980-2015: a systematic analysis for the Global Burden of Disease Study 2015. *Lancet* 388 1459–1544. 10.1016/S0140-6736(16)31012-1 27733281 PMC5388903

[B59] Norman J. M. Handley S. A. Baldridge M. T. Droit L. Liu C. Y. Keller B. C. (2015). Disease-specific alterations in the enteric virome in inflammatory bowel disease. *Cell* 160 447–460. 10.1016/j.cell.2015.01.002 25619688 PMC4312520

[B60] Ostojic S. M. (2018). Inadequate production of H2 by gut microbiota and Parkinson disease. *Trends Endocrinol. Metab.* 29 286–288. 10.1016/j.tem.2018.02.006 29478695

[B61] Parks D. H. Tyson G. W. Hugenholtz P. Beiko R. G. (2014). STAMP: statistical analysis of taxonomic and functional profiles. *Bioinformatics* 30 3123–3124. 10.1093/bioinformatics/btu494 25061070 PMC4609014

[B62] Patro R. Duggal G. Love M. I. Irizarry R. A. Kingsford C. (2017). Salmon provides fast and bias-aware quantification of transcript expression. *Nat. Methods* 14 417–419. 10.1038/nmeth.4197 28263959 PMC5600148

[B63] Peck S. C. Denger K. Burrichter A. Irwin S. M. Balskus E. P. Schleheck D. (2019). A glycyl radical enzyme enables hydrogen sulfide production by the human intestinal bacterium Bilophila wadsworthia. *Proc. Natl. Acad. Sci. U.S.A.* 116 3171–3176. 10.1073/pnas.1815661116 30718429 PMC6386719

[B64] Qian Y. Yang X. Xu S. Wu C. Song Y. Qin N. (2018). Alteration of the fecal microbiota in Chinese patients with Parkinson’s disease. *Brain Behav. Immun.* 70 194–202. 10.1016/j.bbi.2018.02.016 29501802

[B65] Qin J. Li Y. Cai Z. Li S. Zhu J. Zhang F. (2012). A metagenome-wide association study of gut microbiota in type 2 diabetes. *Nature* 490 55–60. 10.1038/nature11450 23023125

[B66] Qureshi I. A. Mehler M. F. (2013). Towards a ‘systems’-level understanding of the nervous system and its disorders. *Trends Neurosci.* 36 674–684. 10.1016/j.tins.2013.07.003 23988221 PMC3818389

[B67] Rehman A. Rausch P. Wang J. Skieceviciene J. Kiudelis G. Bhagalia K. (2016). Geographical patterns of the standing and active human gut microbiome in health and IBD. *Gut* 65 238–248. 10.1136/gutjnl-2014-308341 25567118

[B68] Ribaldone D. G. Pellicano R. Actis G. C. (2018). Inflammation: a highly conserved, Janus-like phenomenon-a gastroenterologist’ perspective. *J. Mol. Med.* 96 861–871. 10.1007/s00109-018-1668-z 29987405

[B69] Sampson T. R. Debelius J. W. Thron T. Janssen S. Shastri G. G. Ilhan Z. E. (2016). Gut microbiota regulate motor deficits and neuroinflammation in a model of Parkinson’s disease. *Cell* 167 1469–1480.e12. 10.1016/j.cell.2016.11.018 27912057 PMC5718049

[B70] Scheperjans F. Aho V. Pereira P. A. Koskinen K. Paulin L. Pekkonen E. (2015). Gut microbiota are related to Parkinson’s disease and clinical phenotype. *Mov. Disord.* 30 350–358. 10.1002/mds.26069 25476529

[B71] Seemann T. (2014). Prokka: rapid prokaryotic genome annotation. *Bioinformatics* 30 2068–2069. 10.1093/bioinformatics/btu153 24642063

[B72] Segata N. Izard J. Waldron L. Gevers D. Miropolsky L. Garrett W. S. (2011). Metagenomic biomarker discovery and explanation. *Genome Biol.* 12:R60. 10.1186/gb-2011-12-6-r60 21702898 PMC3218848

[B73] Smith P. M. Howitt M. R. Panikov N. Michaud M. Gallini C. A. Bohlooly Y. M. (2013). The microbial metabolites, short-chain fatty acids, regulate colonic Treg cell homeostasis. *Science* 341 569–573. 10.1126/science.1241165 23828891 PMC3807819

[B74] Soergel D. A. Dey N. Knight R. Brenner S. E. (2012). Selection of primers for optimal taxonomic classification of environmental 16S rRNA gene sequences. *ISME J.* 6 1440–1444. 10.1038/ismej.2011.208 22237546 PMC3379642

[B75] Song S. J. Lauber C. Costello E. K. Lozupone C. A. Humphrey G. Berg-Lyons D. (2013). Cohabiting family members share microbiota with one another and with their dogs. *Elife* 2:e00458. 10.7554/eLife.00458.018 PMC3628085 23599893

[B76] Sperringer J. E. Addington A. Hutson S. M. (2017). Branched-chain amino acids and brain metabolism. *Neurochem. Res.* 42 1697–1709. 10.1007/s11064-017-2261-5 28417264

[B77] Sun M. F. Shen Y. Q. (2018). Dysbiosis of gut microbiota and microbial metabolites in Parkinson’s disease. *Ageing Res. Rev.* 45 53–61. 10.1016/j.arr.2018.04.004 29705121

[B78] Sun M. F. Zhu Y. L. Zhou Z. L. Jia X. B. Xu Y. D. Yang Q. (2018). Neuroprotective effects of fecal microbiota transplantation on MPTP-induced Parkinson’s disease mice: gut microbiota, glial reaction and TLR4/TNF-alpha signaling pathway. *Brain Behav. Immun.* 70 48–60. 10.1016/j.bbi.2018.02.005 29471030

[B79] Thursby E. Juge N. (2017). Introduction to the human gut microbiota. *Biochem. J.* 474 1823–1836. 10.1042/BCJ20160510 28512250 PMC5433529

[B80] Truong D. T. Franzosa E. A. Tickle T. L. Scholz M. Weingart G. Pasolli E. (2015). MetaPhlAn2 for enhanced metagenomic taxonomic profiling. *Nat. Methods* 12 902–903. 10.1038/nmeth.3589 26418763

[B81] Unger M. M. Spiegel J. Dillmann K. U. Grundmann D. Philippeit H. Burmann J. (2016). Short chain fatty acids and gut microbiota differ between patients with Parkinson’s disease and age-matched controls. *Parkinsonism Relat. Disord.* 32 66–72. 10.1016/j.parkreldis.2016.08.019 27591074

[B82] Wang S. Li N. Zou H. Wu M. (2019). Gut microbiome-based secondary metabolite biosynthetic gene clusters detection in Parkinson’s disease. *Neurosci. Lett.* 696 93–98. 10.1016/j.neulet.2018.12.021 30572101

[B83] Wexler A. G. Goodman A. L. (2017). An insider’s perspective: *Bacteroides* as a window into the microbiome. *Nat. Microbiol.* 2:17026. 10.1038/nmicrobiol.2017.26 28440278 PMC5679392

[B84] Wu M. McNulty N. P. Rodionov D. A. Khoroshkin M. S. Griffin N. W. Cheng J. (2015). Genetic determinants of in vivo fitness and diet responsiveness in multiple human gut *Bacteroides*. *Science* 350:aac5992. 10.1126/science.aac5992 26430127 PMC4608238

[B85] Zhang C. Zhao L. (2016). Strain-level dissection of the contribution of the gut microbiome to human metabolic disease. *Genome Med.* 8:41. 10.1186/s13073-016-0304-1 27098841 PMC4839137

[B86] Zhang H. Yohe T. Huang L. Entwistle S. Wu P. Yang Z. (2018). dbCAN2: a meta server for automated carbohydrate-active enzyme annotation. *Nucleic Acids Res.* 46 W95–W101. 10.1093/nar/gky418 29771380 PMC6031026

[B87] Zhu B. Wang X. Li L. (2010). Human gut microbiome: the second genome of human body. *Protein Cell* 1 718–725. 10.1007/s13238-010-0093-z 21203913 PMC4875195

