# Supplementary figures and images for: Cross-Sectional Study on the Gut Microbiome of Parkinson’s Disease Patients in Central China
Source: Front Microbiol. 2021 Sep 28;12:728479. doi: 10.3389/fmicb.2021.728479 (PMC8506127; doi:10.3389/fmicb.2021.728479)

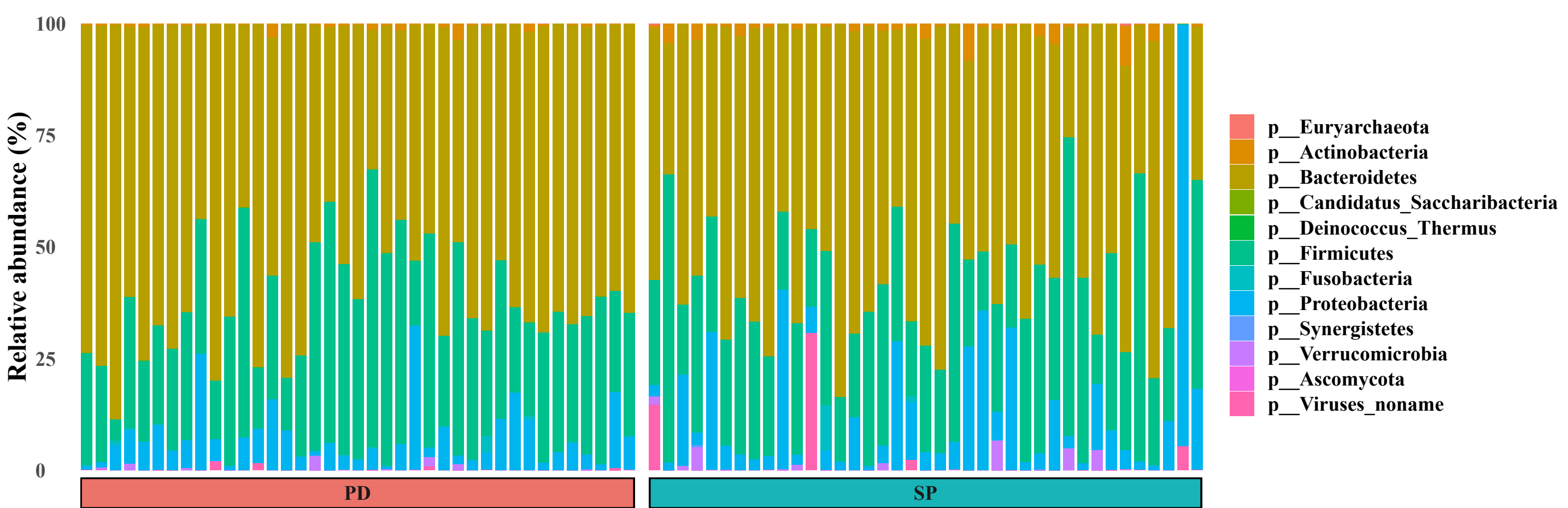

Supplement: Supplementary Figure 1 — Distribution of phyla in all samples. [file Data_Sheet_1.PDF]
